# Understanding levels of engagement and readiness for change in an eHealth sleep program for children with neurodevelopmental disorders

**DOI:** 10.3389/frsle.2024.1455483

**Published:** 2024-09-18

**Authors:** Emily M. Wildeboer, Brooklyn Andrea, Shelly Weiss, Penny Corkum

**Affiliations:** ^1^Department of Psychology and Neuroscience, Dalhousie University, Halifax, NS, Canada; ^2^Division of Neurology, Hospital for Sick Children, University of Toronto, Toronto, ON, Canada

**Keywords:** neurodevelopmental disorders (NDD), pediatric insomnia, sleep, eHealth, behavioral intervention, program engagement and adherence, readiness for change, COVID-19 pandemic

## Abstract

**Background:**

Children with neurodevelopmental disorders (NDD) experience high rates of sleep problems. The *Better Nights, Better Days for Children with Neurodevelopmental Disorders*^*TM*^ *(BNBD-NDD*^*TM*^*)* program is an online intervention for parents of children with NDD who have insomnia/insomnia symptoms. The program has recently undergone a national implementation study (recruitment completed; data collection and analysis ongoing), where parental adherence and engagement are being evaluated. Preliminary results have shown that despite high levels of recruitment, there is less utilization of the program than the research team expected. Parental engagement may have been impacted by participants' motivation and readiness for change, as well as indirectly by the COVID-19 pandemic. The objective of the current study is to better understand engagement with the *BNBD-NDD*^*TM*^ program concerning parental motivation and readiness for change, while considering the possible impacts of COVID-19.

**Methods:**

Parents of children with NDD (*n* = 18) who were enrolled in the *BNBD-NDD*^*TM*^ program for a minimum of 4 months completed exit interviews using a researcher-generated, semi-structured interview guide. During the interview, participants were asked about their engagement in the program, perspectives on their own readiness for changing their children's sleep, and the impact of COVID-19 on their engagement. Data were analyzed following an inductive content analysis approach.

**Results:**

Several categories of data were generated that explain levels of engagement, including: (1) severity of sleep problems; (2) motivation for change; (3) previous strategies for sleep; (4) confidence in the program; (5) sacrifices made to change sleep; (6) maintenance of change; (7) experience with levels of support provided; and (8) impact of the COVID-19 pandemic.

**Conclusion:**

Parents identified several factors related to their readiness for change as contributors to their engagement level in the *BNBD-NDD*^*TM*^ program. The COVID-19 pandemic had varied impacts on engagement for participants in this sample. Understanding parents' engagement levels within the *BNBD-NDD*^*TM*^ eHealth program related to their motivation and readiness for change is crucial to optimize uptake and adherence to the program, improve the program's implementation and sustainability, and continue to help children with NDD to sleep better.

## 1 Introduction

Sleep problems are one of the most reported concerns in children, affecting ~30% of children (Esposito et al., 2019; Mindell et al., 2006). Pediatric insomnia includes frequent and chronic difficulty with falling asleep and/or staying asleep (Mindell et al., 2006), and can have numerous effects on both physical, mental, and cognitive functioning (e.g., increased sleepiness, fatigue, and poorer emotional regulation; Bub et al., 2011; Reid et al., 2009; Sadeh, 2007). Children with neurodevelopmental disorders (NDD) experience an even greater rate of pediatric insomnia (Belli et al., 2022; Didden and Sigafoos, 2001; Shelton and Malow, 2021; Tietze et al., 2012), and there is evidence that they may be more vulnerable to the negative consequences of poor sleep than their neurotypical counterparts (Sadeh, 2007; Shelton and Malow, 2021; Vriend et al., 2011).

The first-line treatment of pediatric insomnia for both neurotypical children and those with NDD focus on behavioral strategies. As behavioral factors often contribute to insomnia (e.g., inconsistent bed and waketimes, increased use of electronics at bedtime), behavioral interventions are typically effective in mitigating sleep concerns (Blackmer and Feinstein, 2016; Heussler, 2016; Jan et al., 2008; Mindell et al., 2006; Vriend et al., 2011). There is growing evidence for the effectiveness of behavioral interventions for various concerns for both neurotypical children and those with NDD (Meltzer et al., 2021) and as such, there is a need for behavioral programs to be created and adapted specifically for insomnia in neurotypical children and children with NDD.

One example of a behavioral program targeted at neurotypical children with sleep concerns is *Better Nights, Better Days for Typically Developing Children*^*TM*^ *(BNBD-TD*^*TM*^; Corkum et al., 2018). This program is an online intervention targeted at parents of children ages 1–12 years with pediatric insomnia. The *BNBD-TD*^*TM*^ program is empirically supported through rigorous scientific testing, including a national randomized controlled trial (RCT) and usability and implementation studies (Corkum et al., 2018; Jia et al., 2023; Orr et al., 2019), and high levels of user satisfaction have been reported by parents who have completed the program. The *BNBD-TD*^*TM*^ program has since been expanded for babies (age 6–12 months) and youth (i.e., adolescents and university students).

While the *BNBD-TD*^*TM*^ program provides high quality behavioral intervention for pediatric insomnia in neurotypical children, one gap identified in its development and implementation was the application to children with NDD. It is well-established that there is increased risk for pediatric insomnia in this group, and there are also several unique sleep-related challenges for families with children with NDD, including higher frequency of bedtime resistance and nighttime awakenings, less napping, greater NDD symptom severity, and greater treatment resistance. As such, it was imperative to modify *BNBD-TD*^*TM*^ to better suit the needs of children with NDD. Research has also shown that parents of children with NDD find eHealth interventions to be acceptable and usable (Tan-MacNeill et al., 2020a,b, 2021). As such, the *Better Nights, Better Days for Children with Neurodevelopmental Disorders (BNBD-NDD)*^*TM*^ program was developed with specific focus on children with Attention Deficit Hyperactivity Disorder (ADHD), Autism Spectrum Disorder (ASD), Cerebral Palsy (CP), and Fetal Alcohol Syndrome Disorder (FASD). This program is rooted in empirical literature, healthcare professional expertise and consensus, the parent/family perspective, and has been developed and evaluated through varied scientific methods, including exit interviews, usability testing, and a Delphi study (Ali et al., 2018; Ilie et al., 2023; Rigney et al., 2018; Tan-MacNeill et al., 2020a,b; Vaughan et al., 2022). The *BNBD-NDD*^*TM*^ program is an online, self-guided program for parents/caregivers of children with NDD and insomnia. There are five modules that teach parents various sleep strategies with a specific focus on the impact of NDD on sleep. The program is estimated to take about 5–10 weeks to complete. Preliminary findings from a recent RCT (presented by Vaughan et al., 2022) have demonstrated a high level of parent satisfaction with the program, strong implementation success, and parental reports of effectiveness.

Preliminary findings from a recent study on barriers and facilitators to engagement in the *BNBD-NDD*^*TM*^ program during the RCT (Ilie et al., 2023) have shown lower engagement in and underutilization of content and supports in the program than expected. Participant feedback elicited in exit interviews after the RCT included suggestions around scheduling more reminders and adding personalized and tailored evidence to specific participant concerns (Ilie et al., 2023). Participants also expressed the need for a variety of supports to be made available for participants, such as online coaching and/or virtual communities/hubs for information (Ilie et al., 2023). As result of these findings from the RCT, the *BNBD-NDD*^*TM*^ program was modified, and additional supports were added. These supports included access to an online coach, a research team member trained in the *BNBD-NDD*^*TM*^ program who could provide additional information and support as requested, and a virtual hub, which is an online portal that includes additional resources, webinars, and information from a number of sleep experts. The program is currently undergoing a national implementation study (recruitment completed; data collection and analysis ongoing). While there has been a good level of recruitment, there has been a lower-than-expected level of engagement from participants. In order to optimize the effects of the *BNBD-NDD*^*TM*^ program, it is prudent to explore participant perspectives on the new adaptations to the program, as well as understand parental adherence in the program and consider why there has been a lower rate of engagement than expected thus far.

There are many different factors that can impact one's engagement and adherence in parent-focused interventions such as the *BNBD-NDD*^*TM*^ program, including their motivation to participate fully in the intervention. Based on the Stages of Change Model, a well-established, transtheoretical model of behavior change (Prochaska et al., 1992; Zimmerman et al., 2000), individuals engaging in interventions often fall into a particular stage of change. When patients do not consider change at all (i.e., are in denial, see no problems, or give up on change), they are considered in the precontemplation stage. In the contemplation stage, patients begin thinking about the pros and cons of change but are ambivalent toward taking steps to make these changes. The preparation stage occurs when patients prepare to make a specific change and begin to experiment with small changes; however, they are not yet committed to full action. The action stage is the ideal stage for patients undergoing an intervention, as in this stage they demonstrate a desire for lifestyle change and put action toward achieving their goals. Lastly, as patients begin to incorporate these changes long-term, they progress into the maintenance and relapse prevention stage (Prochaska et al., 1992). In relation to the *BNBD-NDD*^*TM*^ program, it is plausible to assume that engagement of the participants in the program may be related to the different stages of change of the participating parents.

In order to better understand the levels of engagement of participants in the *BNBD-NDD*^*TM*^ implementation study, as well as examine the impact of readiness for change on engagement and evaluate the use of newly developed supports (i.e., access to an online coach and/or a virtual hub), a qualitative study was conducted with participants of varying levels of engagement (i.e., Clinical Engagement [3–5 sessions completed], Non-clinical Engagement [1–2 sessions completed], and No Engagement [< 1 session completed]). Data were collected through virtual semi-structured interviews and analyzed through inductive content analysis (Elo and Kyngäs, 2008; Vears and Gillam, 2022). Data were coded based on the engagement levels of participants, their randomized level of support in the implementation study (i.e., self-guided, access to an online coach, and online coach plus access to the virtual hub), and factors related to readiness for change according to the Stages of Change model (Prochaska et al., 1992).

This study had four objectives. The first objective was to understand the factors that contributed to different levels of engagement among participants (i.e., Clinical Engagement, Non-clinical Engagement, and No Engagement). The second objective was to explore the relationship between readiness for change and engagement in the *BNBD-NDD*^*TM*^ program according to the Stages of Change model (Prochaska et al., 1992; Zimmerman et al., 2000). The third objective was to explore the use of and satisfaction with levels of support in the *BNBD-NDD*^*TM*^ program related to engagement level. Lastly, as the *BNBD-NDD*^*TM*^ implementation study was largely conducted during the COVID-19 pandemic time period, the fourth objective was to understand the possible impacts of the pandemic on engagement and/or use of supports in the program.

## 2 Methods

### 2.1 Participants

Eligible participants recruited for this study had previously consented to participate in the *BNBD-NDD*^*TM*^ implementation study, were living in Canada, had been enrolled in the *BNBD-NDD*^*TM*^ program for at least 4 months, and had not asked to be withdrawn from the implementation study. Although all participants had been enrolled in the program by the researchers, they were not required to have started or completed the program to participate in the current study.

Of the 112 participants who met inclusion criteria and were contacted to participate in this study, 26 consented to participate, and 18 parents/caregivers completed semi-structured qualitative interviews. The eight participants who consented but did not participate had either not scheduled an interview or did not attend their scheduled interview. Of the parents who did attend interviews, six participants were in the Clinical Engagement group, meaning that they had completed at least three of five sessions of the program. These participants were considered Clinically Engaged as they had completed at least the first three sessions; as such, they had received the core intervention for addressing difficulties with initiating and maintaining sleep. It is expected that this information would have been enough intervention for these parents to have made clinically significant changes and/or improvements in their child's sleep. Five participants were in the Non-clinical Engagement group, meaning that at the time of the current study, they had completed only one or two sessions of the program. While engaged, these participants were considered Non-clinically Engaged as they did not receive enough of the core intervention strategies to address difficulties initiating and maintaining sleep; as such, it would be unlikely that they had experienced enough of the program to see clinically significant changes or improvements. Seven participants were in the No Engagement group, meaning that at the time of the current study, they had never accessed the program (*n* = 1), or accessed it but did not complete the first session (*n* = 6).

Participants were further stratified based on their randomized level of support from the *BNBD-NDD*^*TM*^ implementation study. Five participants had been randomized to Level 1, the self-guided program (i.e., no additional support). Seven participants had been randomized to Level 2, meaning they had access to an online coach for support, and six had been randomized to Level 3, meaning they had access to the online coach as well as the virtual hub. See Table 1 for a breakdown of the participant engagement and level of support group representation.

**Table 1:** Participant engagement and level of support group representation.

	**Clinical Engagement**	**Non-clinical Engagement**	**No Engagement**	**Total**
Level 1 support	1	2	2	5
Level 2 support	3	1	3	7
Level 3 support	2	2	2	6
Total	6	5	7	18

Participants were parents of children diagnosed with a variety of neurodevelopmental disorders. Most children were male (77.79%) and were diagnosed with ADHD (55.56%), followed by ASD (38.89%), other NDD (16.67%), CP (11.11%), and FASD (5.56%). Children were between the ages of 6 and 13 years at the time of parent participation in the program (*M* = 9.00 years, *SD* = 1.97 years).

### 2.2 Materials

#### 2.2.1 Demographic questionnaire

Participants completed a demographics questionnaire during the broader *BNBD-NDD*^*TM*^ implementation study. This questionnaire contained 32 items related to demographic and socio-economic information about the participant, their spouse/partner, and the child with the NDD who the focus of the intervention was on. For the purposes of this study, only data relevant to the age, sex, and diagnosis of the child was reported.

#### 2.2.2 Semi-structured interview guide

The interview guide included 13 researcher-generated, open-ended questions related to participants' engagement in the *BNBD-NDD*^*TM*^ program, retrospective perspectives on their readiness for change (i.e., experiences related to severity of sleep problems, motivation for change, previous strategies, confidence in the program, sacrifices made, and maintenance of change), their experience with provided levels of support (i.e., self-guided, online coach, and/or virtual hub), and the impact of the COVID-19 pandemic on their engagement in the program. Questions related to levels of support varied slightly based on the support level participants had been randomized to in the *BNBD-NDD*^*TM*^ implementation study. Participants were also asked if they had any other questions, comments, or feedback regarding the *BNBD-NDD*^*TM*^ program.

### 2.3 Procedure

Once participants had been enrolled in the *BNBD-NDD*^*TM*^ program for at least 4 months, they were contacted to participate in the survey regardless of their current engagement level. All eligible participants were contacted via an initial recruitment email and received a maximum of three email and two phone reminders. Eligible participants were provided with a link to a secure online survey platform, the Research Electronic Data Capture (REDCap; Harris et al., 2019), which included a letter of information, consent form, and interview appointment scheduling tool. Interested participants provided written consent on the electronic form and were subsequently contacted by the research team to finalize their interview time. Participants were also sent reminder emails 24 and 2 h before their interview.

During the interview, relevant consent information was summarized by the interviewer (EMW) and participants indicated verbal consent to participate. Virtual interviews were conducted without video and were audio-recorded and transcribed using the web-based Microsoft Teams software. Each interview was conducted by the first author (EMW) with a volunteer notetaker present. Interview duration ranged from 14 to 30 min (*M* = 21 min, *SD* = 0.20 min). Participants were given a $25 Amazon gift card as an honorarium for participating in the study after their interview concluded.

### 2.4 Data analysis

Participant responses were analyzed for each question individually following an inductive content analysis approach (Elo and Kyngäs, 2008; Thomas, 2006; Vaismoradi et al., 2013), using QSR International's NVivo 12 analysis software (Lumivero, 2023). The analysis was conducted with two coders (EMW and BA; Elo et al., 2014). First, both coders reviewed each transcript to become familiar with the data. Subsequently, the first author (EMW) reviewed each transcript to develop initial codes and create codebooks based on each engagement level. Then, using line-by-line coding, both coders each coded 33% of the responses (i.e., two transcripts from each engagement level) and compared their coding. The coders then met to discuss and refine the codes further. Following final development of the codebooks, the first author (EMW) grouped like codes. These groupings were then abstracted into higher-order categories and a primary description for each was developed. To ensure consistency and accuracy throughout this process, the two coders met frequently to discuss the codes and abstraction of categories and maintained a detailed audit trail of analytical decisions. Codes, categories, and any discrepancies were discussed with a senior member of the research team (PC) to build rigor and trustworthiness. Lastly, select quotations were identified to indicate the conformability and trustworthiness of results (Graneheim and Lundman, 2004).

## 3 Results

Participant responses to each section of the interview were analyzed based on engagement group using inductive content analysis. Data are presented below beneath each of the four research objectives of this study.

### 3.1 Research objective 1: engagement levels

Participants were asked about their engagement levels in the program and any barriers to engagement. Sample quotations related to engagement levels can be found in Table 2.

**Table 2:** Participant quotations related to engagement levels.

	**Sample quotations**
**Clinical Engagement**	
High level of engagement	“… once I started, I did finish it till the end.” (Participant 018, Clinical Engagement group)
Engagement varied over time	“… instead of moving on, I'd go back, and I tried a couple different ones [strategies] that I didn't think would work, but then realized, wait a minute, they actually… do work better than I thought they would.” (Participant 013, Clinical Engagement group)
**Non-clinical Engagement**	
Some engagement	“I've done 2 sessions and now I'm just taking a break to make some of the changes that session 2 recommended.” (Participant 020, Non-clinical Engagement group)
Content as barrier	“Before the program, we had already been doing a lot of the stuff… it still took a lot of time to go through it and I didn't really gain anything because I was already doing all those things.” (Participant 006, Non-clinical Engagement group)
Time as barrier	“Time was… the main barrier in terms of completing it… I had all intentions of finishing it… for me, it was 100% time. Like our life, just personal reasons… I just couldn't devote that much time to do it.” (Participant 006, Non-clinical Engagement group)
	“I think the changes for our family are big for us… I have to make sure we've got this session [2] stuff down… before I try Session 3.” (Participant 020, Non-clinical Engagement group)
**No Engagement**	
No engagement	“We haven't done the first [session] yet because it takes a 2 h time block to get through, and so that's where we kind of fell off track.” (Participant 024, No Engagement group)
Content as barrier	“You don't… get the option to start where you need to start… it starts with assuming your child has trouble falling asleep, so you have to go through the first session which teaches you how to get your kid to fall asleep. But that has never been an issue for us.” (Participant 012, No Engagement group)
Time as barrier	“I think things just got busy around here and I just couldn't make the time.” (Participant 012, No Engagement group)
	“You know, life… for moms with neurodiverse children and multiple children, it's busy. Like it's so busy that I just have to find time to sit down and log in and get my mind around starting the program… that's what prevented me from starting for a long time.” (Participant 019. No Engagement group)
Lack of motivation	“I didn't feel the motivation to get it started.” (Participant 014, No Engagement group)

#### 3.1.1 Clinical Engagement

Six participants were in the Clinical Engagement group. Most participants reported that they tried to implement most of the strategies from the program into their daily lives, and some also explained that they went back through past sessions when necessary to get more information. Two participants, while still clinically engaged, also identified that their engagement had varied over time. One participant described being more engaged during the first few sessions of the program compared to the last few, while another identified that they went back to previous sessions several times to rewatch videos and/or reread information.

#### 3.1.2 Non-clinical Engagement

Five participants were in the Non-clinical Engagement group. When asked what impacted their engagement, most participants (*n* = 4) described that time and external circumstances (e.g., unforeseen circumstances) were a barrier to completion, while two participants further identified the amount (i.e., too much information for parents to sort through) and relevance of the program's content as barriers.

#### 3.1.3 No Engagement

Seven participants were in the No Engagement group. One participant did not start the program at all, while the rest did not finish the first session. Participants in this group identified several barriers to their engagement, including that the program required a high level of time and resource commitment (*n* = 5), which was not always feasible, that not all the content of the program appeared relevant (*n* = 1), and an overall lack of motivation for engagement (*n* = 1).

### 3.2 Research objective 2: readiness for change

Participants were asked a series of questions related to aspects of their pre-intervention readiness for change and motivation to participant in the *BNBD-NDD*^*TM*^ program. Six categories of data were generated and are elaborated upon below. Sample quotations supporting each summary can be found in Table 3.

**Table 3:** Participant quotations related to readiness for change.

	**Sample quotations**
**Severity of sleep problems**	
Impact on child	“It really makes a big difference in terms of how she can cope with the day, how available she is to learning, to social situations, those kinds of things.” (Participant 001, Non-clinical Engagement group)
	“He would feel a lot of stress and anxiety around sleep because he knew that it was really hard for him to fall asleep, and every night, he would know what was coming.” (Participant 002, Clinical Engagement group)
	“Our son obviously wasn't well-rested, so he wasn't well-behaved during the day… it was hard for him to sit through class and pay attention. He disturbed other children and their learning.” (Participant 018, Clinical Engagement group)
	“We were concerned about the amount of sleep that he was getting, and considering he has ADHD, I just felt like it created this negative cycle of not enough sleep and then it affecting his behavior in the morning.” (Participant 019, No Engagement group)
Impact on family	“I basically had no time to do anything at the end of the day because I would be with him. He would take so long to go to sleep at night.” (Participant 013, Clinical Engagement group)
	“It impacted the sleep of my other children… waking up the other kids, and then they were grumpy because they didn't sleep well.” (Participant 018, Clinical Engagement group)
	“We were extremely sleep deprived and really exhausted on a daily basis… it was a lot of stress to try to get him to wake up for school, try to get anywhere on time. It was very problematic for all of us for sure.” (Participant 020, Non-clinical Engagement group)
	“It's affected my memory… I'm not who I used to be… I don't feel fully functional anymore. It's really affected us.” (Participant 021, No Engagement group)
**Motivation for change**	
High motivation	“I was desperate for a change and willing to put in the work for this program to try and see if it would help.” (Participant 002, Clinical Engagement group)
	“It was definitely a high priority… I was very motivated, very excited when I saw the ad for the program.” (Participant 020, Non-clinical Engagement group)
Lack of success with previous strategies	“We tried a variety of things… but it just seemed like it wasn't really working.” (Participant 003, Clinical Engagement group)
	“We had, a number of years ago, tried a behavioral consultant who came in and evaluated our routine and gave some suggestions, but I think both my husband and I just couldn't stick to whatever that routine was.” (Participant 017, No Engagement group)
Long-lasting problems	“… this is always how he's been. I don't know what it would be like if he had a full 9 or 8 h every night. I don't know what that would look like.” (Participant 021, No Engagement group)
Uncertainty about how to make change	“Motivated, yes. And then also at the same time, not knowing how I'm gonna get the energy to do it.” (Participant 021, No Engagement group)
**Previous strategies**	
Several past strategies for sleep	“This is a long list… nightly baths… different kinds of sheets… propping up the bed, I tried vibrating things, sound machines, music, blackout curtains, massages… rocking her to sleep, firmer method of letting her cry it out, lights, no lights… I really feel it was very, very, very exhaustive.” (Participant 001, Non-clinical Engagement group)
	“We've seen a psychologist, behavior therapist, a sleep physician… sleep studies and melatonin studies… lots of professionals have been seen about sleep, occupational therapy. Basically, I think anybody you could see.” (Participant 009, Clinical Engagement group)
Lack of success with previous strategies	“Some of those other options were not long-term fixes… either not a lot of long-term success or options that really aren't going to be viable long-term or are contradictory with other types of supports and resources that the kiddo needs.” (Participant 009, Clinical Engagement group)
	“… I guess it [melatonin] works for some people and not others. But for him, it makes him more anxious and then sleepy at the same time.” (Participant 021, No Engagement group)
	“… we would make an effort to wake him up early, and he would just basically be sleeping standing up.” (Participant 022, No Engagement group)
**Confidence in program**	
Cautious optimism	“… a little skeptical but also hopeful. So, it's somewhere in between.” (Participant 025, Clinical Engagement group)
Confidence about structure of program	“What attracted me to it was… the feeling that somebody was in control of this process, it wasn't just me reading a book and then trying to implement it by myself and failing… it was more structured.” (Participant 003, Clinical Engagement group)
	“I also thought because it was aimed for more children that weren't necessarily your neurotypical child, that would also be a benefit.” (Participant 013, Clinical Engagement group)
	“I had high hopes… because the program was targeting, in our case, children or families who are dealing with ADHD. So, I was much more hopeful, and I just thought, okay, well, they've put more effort into something that applies much more to us.” (Participant 020, Non-clinical Engagement group)
Low confidence	“I wouldn't say I had a huge confidence that there was something in there that was gonna help because I felt like we were already doing all the things.” (Participant 006, Non-clinical Engagement group)
	“We had no confidence at all that this would ever improve.” (Participant 012, No Engagement group)
**Sacrifices**	
Time as sacrifice	“Finding time was definitely a problem for us… the last couple of years, we have not had much free time.” (Participant 017, No Engagement group)
	“Time would have been the big sacrifice.” (Participant 024, No Engagement group)
Routine as sacrifice	“I had to make sure that I was committing the time… I did have to change the way I went about my day being more conscious of the time and making sure that I was able to start the routine.” (Participant 013, Clinical Engagement group)
Willingness to make sacrifices	“Definitely, yeah, because it's for my child's health and for our whole family's wellbeing.” (Participant 018, Clinical Engagement group)
	“I think certainly we would have been willing to financially invest if that was something that was required and there was proven benefit.” (Participant 024, No Engagement group)
**Maintenance of change**	
Cautious optimism	“I would say as confident as coming into it so, cautiously optimistic.” (Participant 009, Clinical Engagement group)
Low confidence	“I definitely was not sure at all that we'd be able to do this one.” (Participant 017, No Engagement group)
	“… my biggest concern is that we will struggle to maintain it [change].” (Participant 020, Non-clinical Engagement group)

#### 3.2.1 Severity of sleep problems

Across the three engagement groups, participants varied in their reports regarding the severity of their child's sleep problems. The highest severity was reported in the Clinical Engagement group compared to the other groups; however, all three groups identified similar impact of these problems on the daily functioning of the child and family. For example, reported impacts of sleep problems on children included poor mood, inattention, difficulty with social relationships, and physical wellbeing (e.g., energy levels). Across all three groups, sleep problems were also reported to have an impact on the broader family functioning, including on parent and sibling sleep, energy levels, and routines.

#### 3.2.2 Motivation for change

When asked about motivation levels regarding making changes in sleep problems, participants in the Clinical Engagement group tended to report high levels of motivation to make change compared to the Non-clinical and No Engagement groups. In both the Clinical and Non-clinical Engagement groups, several participants identified a history of long-lasting problems and unsuccessful previous strategies for sleep management as motivators for change (*n* = 11), whereas in the No Engagement group, some participants reported an uncertainty about how to actually make change (*n* = 2).

#### 3.2.3 Previous strategies

When asked about previous strategies used to target sleep behaviors, most participants, regardless of engagement group, described several past efforts, including sleep studies (i.e., polysomnography), medication, and consultation with various professionals. Further, regardless of engagement group, participants indicated that these previous strategies were largely unsuccessful, ultimately leading to participants pursuing the *BNBD-NDD*^*TM*^ program. Two participants in the No Engagement group reported that they had not tried any previous strategies and that the *BNBD-NDD*^*TM*^ program was the first sleep intervention they pursued.

#### 3.2.4 Confidence in program

Participants' confidence in the ability of the *BNBD-NDD*^*TM*^ program to help their child's sleep problems varied significantly across and within engagement groups. Many participants in the Clinical and Non-clinical Engagement groups (*n* = 9) identified cautious optimism and mid-level confidence due to the program's structure (e.g., having several sleep experts from multidisciplinary fields) and focus on NDD; however, other participants (*n* = 5) described frustration and lack of success with other sleep-related efforts as contributors to lower confidence in the *BNBD-NDD*^*TM*^ program. Some participants in the No Engagement group (*n* = 3) also reported low confidence or uncertainty about the *BNBD-NDD*^*TM*^ program.

#### 3.2.5 Sacrifices

Participants across the engagement groups had varied responses related to making sacrifices to improve sleep problems in children. In all three groups, time and routine were identified as sacrifices by some parents (*n* = 11), while others did not identify any sacrifices (*n* = 6). Further, across all groups, some participants expressed that they would have been willing to make sacrifices if necessary (*n* = 7).

#### 3.2.6 Maintenance of change

Participants in all three engagement groups expressed varying levels of confidence that the program would help maintain changes over time. In the Clinical and Non-clinical Engagement groups, some parents felt like the program would be helpful and identified cautious optimism (*n* = 8), while others lacked confidence in the program's ability to foster long-lasting change (*n* = 4). In comparison, participants in the No Engagement group reported lower levels of confidence that the *BNBD-NDD*^*TM*^ program would help them maintain changes over time (*n* = 4), though one participant indicated that they had not really thought about the long-term impacts of the program.

### 3.3 Research objective 3: levels of support

Participant responses were categorized based on their randomized level of support (i.e., self-guided, online coach, and/or virtual hub). Sample quotations can be found in Table 4.

**Table 4:** Participant quotations related to levels of support.

	**Sample quotations**
**Level 1: self-guided program**	
Would have used extra supports	“I think it definitely could have helped the whole process because it's really overwhelming and sometimes just to be able to bounce an idea off somebody, is just a little bit easier.” (Participant 001, Non-clinical Engagement group)
	“I'm always of the philosophy, the more support, the better. So, if there was something additional available to me, that would have been helpful.” (Participant 025, Clinical Engagement group)
Supports may have impacted engagement	“If there was that commitment to another person and to another individual, I think we definitely would have made more time to go through it. There would have been more motivation.” (Participant 017, No Engagement group)
**Level 2: online coach**	
Coach was helpful	“I was kind of confused, but they were able to clarify… so then I could proceed.” (Participant 002, Clinical Engagement group)
Coach was unnecessary	“I guess I didn't really feel like I needed it because it felt pretty straightforward.” (Participant 003, Clinical Engagement group)
Unaware of coach	“I never used it because I forgot I was in that one [group] and also didn't know how to do it.” (Participant 013, Clinical Engagement group)
	“I haven't thought about emailing for support really at all. I've been super overwhelmed by some of what we have to do, but… it just didn't occur to me to email and say I'm having a hard time.” (Participant 020, Non-clinical Engagement)
Would have used virtual hub	“I probably would have went in and checked it out for sure.” (Participant 013, Clinical Engagement group)
	“I'm reading about my son all the time… I like to get as much information as I can, so I probably would have found that interesting for sure.” (Participant 021, No Engagement group)
**Level 3: online coach and virtual hub**	
Coach was unnecessary	“… the answer they gave, I totally get where they're coming from. And I was like, yeah, that's in principle what's supposed to happen. And is it gonna work in this case?” (Participant 009, Clinical Engagement group)
Unaware of coach	“I wasn't aware that my group had the option to access it.” (Participant 018, Clinical Engagement group)
Virtual hub was helpful	“I watched a few videos on there that had information about specific things I was interested in.” (Participant 006, Non-clinical Engagement group)
Virtual hub was not helpful	“Some of it was a lot of the same kind of information that you would get through the program. So, I didn't really fully understand what the purpose of the virtual hub was.” (Participant 006, Non-clinical Engagement group)

#### 3.3.1 Level 1: self-guided program

All participants who were randomized to Level 1 (*n* = 5) predicted that they would have used the coach and virtual hub if they had access to them and may have found them as helpful resources if they provided extra content. The two Level 1 participants who were in the No Engagement group also speculated that having these additional supports may have increased their commitment to the program.

#### 3.3.2 Level 2: online coach

Participants who were randomized to Level 2 (*n* = 7) reported varied experiences when accessing the online coach. Only one participant identified that they contacted the coach and expressed that they thought the coach was helpful and provided good support. In contrast, the other participants did not use the coach for support as they did not feel it was necessary. Further, some participants identified that they were unaware they had access to a coach (*n* = 3). When asked about potential use of the virtual hub, all participants identified potential positive benefits; however, two participants expressed that they did not feel confident the virtual hub would have improved success with the program.

#### 3.3.3 Level 3: online coach and virtual hub

Participants who were randomized to Level 3 (*n* = 6) also reported varied experiences with the online coach and virtual hub. Only one participant reported that they had accessed the online coach, and explained that while they found the support well-intentioned, it was not particularly helpful or relevant. The other five participants expressed that they did not realize they had access to the coach; however, some predicted that they would have made use of the coach if aware (*n* = 4). In terms of the virtual hub, there were also mixed feelings. Some participants expressed that they found the virtual hub helpful and informative (*n* = 2), while the others thought it was too repetitive, unappealing, and/or inactive (*n* = 4), ultimately leading to a lack of use.

### 3.4 Research objective 4: impact of COVID-19 pandemic

There was a wide array of perspectives about the impacts of the COVID-19 pandemic on engagement in the *BNBD-NDD*^*TM*^ program. The majority of participants did not identify any impact of the pandemic on their engagement in the program (*n* = 10). Of those who did identify impacts *(n* = 8), several participants (*n* = 5) described positive benefits of the COVID-19 pandemic, including that the online format was very convenient, which helped improve their flexibility and commitment to the program, and that the pandemic actually contributed to sleep improvements as well. One participant described that the pandemic helped increase their awareness about sleep due to the change in routines. Some participants (*n* = 3) did identify negative impacts, including the contribution to long-lasting illness, which subsequently impacted engagement in the program (*n* = 1), as well as challenges related to routines and scheduling (*n* = 2). One participant predicted that the program may have been more helpful to them during the height of the COVID-19 pandemic, compared to participation once restrictions were lifted and routines fell back into place. Sample quotations can be found in Table 5.

**Table 5:** Participant quotations related to the impact of COVID-19 pandemic.

	**Sample quotations**
No impact identified	“I don't know that COVID impacted my use of the program at all… I feel like it didn't really change a whole lot.” (Participant 013, Clinical Engagement group)
	“No, because it was an online program… I don't think COVID-19 changed the impact of that at all.” (Participant 014, No Engagement group)
Positive impact on flexibility and commitment	“With reduced activities and outings, it gave me more time to devote to participating in the program. The pandemic allowed us the time to work through the program with our son.” (Participant 002, Clinical Engagement group)
	“I think COVID forced us all to be able to be more flexible about when and how we work, so I think that was a helpful thing.” (Participant 009, Clinical Engagement)
Positive impact on sleep	“… perhaps more awareness at how things had deteriorated with my son's sleep… our habits would have gotten worse with the pandemic.” (Participant 002, Clinical Engagement group)
	“It helped because we were able to get him to sleep at home for 2–3 h in the day due to the lack of sleep at night.” (Participant 023, Non-clinical Engagement)
Negative impact on illness	“The ongoing COVID-19 pandemic situation had a strong influence in my participation. My child likely has long COVID… and her ongoing health concerns heavily affected her sleep. I think if I had engaged with the program at a time when she was not affected with this medical issue… I could have engaged in the program better.” (Participant 001, Non-clinical Engagement group)
Negative impact on routines	“… our household schedules, just changing every few months… just add different stressors. I feel like if we had a more consistent schedule… or things weren't constantly changing… there would have been more time to just focus.” (Participant 017, No Engagement group)

## 4 Discussion

The purpose of this study was to examine participants' engagement and readiness for change in the *BNBD-NDD*^*TM*^ program and their use of the provided virtual supports in the context of the COVID-19 pandemic. Overall, results of this study showed that the *BNBD-NDD*^*TM*^ program was helpful and well-received by motivated, engaged parents (i.e., Clinical Engagement group). Further, these parents did not appear to find the online coaching or virtual hub particularly helpful or necessary; rather, the program itself seemed like it was enough for them. In contrast, parents who were less motivated and engaged (i.e., Non-clinical and No Engagement groups) reported more barriers to their engagement and indicated a higher desire for more or improved supports.

The first research objective was to understand the factors that contributed to different levels of engagement among participants. Participants in the Non-clinical and No Engagement groups (i.e., those who participated in < 3 sessions of the program) tended to report more barriers to engagement in the program than those in the Clinical Engagement group. These barriers included time, external circumstances, perception of the program's content as irrelevant or excessive, and an overall lack of motivation. While some participants in the Clinical Engagement group did identify these same factors as challenges (i.e., a large time commitment), they appeared more willing/able to make the sacrifices needed to engage in the program.

These findings related to engagement are not particularly surprising given research about treatment adherence in eHealth interventions. Low levels of treatment adherence and engagement, and high levels of attrition, are common in many eHealth interventions (Kelders et al., 2012; Michie et al., 2017). Retention rates of eHealth interventions are often only about 50% and tend to decrease significantly over time (Oakley-Girvan et al., 2021). Various factors can impact engagement and adherence, such as scheduling and time-management issues, forgetfulness, psychiatric comorbidities, behavioral problems, and low motivation for change (Gearing et al., 2014). It is possible that some of these factors were at play in the current study; in particular, participants tended to mention time and logistical barriers as contributors to a lack of motivation.

The second research objective was to examine participants' engagement levels as they related to readiness for change according to the Stages of Change model (Prochaska et al., 1992). Based on results of this study, it appears that participants in the Clinical Engagement group can be classified as in the action stage of behavior change—in other words, they demonstrated a desire for change, put action toward achieving their goals, and were willing to make sacrifices to engage in the program. In contrast, participants in the Non-clinical and No Engagement groups did not quite demonstrate the same level of desire, motivation, or action. More specifically, participants in the Non-clinical Engagement group can be classified in the preparation stage of the Stages of Change model, meaning that they took some steps toward change (e.g., participating in 1–2 sessions of the program); however, they were not yet committed to full action as required for the action stage. Participants in the No Engagement group can be classified in the contemplation stage, meaning that they likely had thought about the pros and cons related to change; however, did not yet take steps toward making change by participating in any sessions of the program. None of the participants in the current study would be classified in the precontemplation stage, as they did, at minimum, at least consider change by consenting to participate in the *BNBD-NDD*^*TM*^ program. Further, none of the participants would be classified in the maintenance stage yet, as they are presumably still working toward incorporating these changes long-term. It is likely that some participants in the Clinical Engagement group may progress to the maintenance stage given their level of motivation and commitment to the program if they continue to practice change and prevent relapse.

While it is important to understand where the participants in the current study are classified in terms of the stages of change, it is even more crucial to consider how they might move from one stage to the next (i.e., from contemplation to action; or from No or Non-clinical Engagement to Clinical Engagement). According to the Stages of Change model, one of the primary factors that impacts one's ability to progress from one stage to the next is decisional balance. Decisional balance is the evaluation of the benefits and costs of one's current behavior compared to potential changed behavior (Prochaska et al., 1992). Research shows that if the evaluation of the benefits and costs of changed behavior results in a perception of more costs than benefits, an individual is most likely in the precontemplation stage (i.e., considering change but not yet taking steps toward it). When the evaluation results in more benefits than costs, an individual is likely in the contemplation stage (i.e., ready to act, though not quite yet engaging in action; Prochaska et al., 1992). As the benefits continue to increase and the individual moves past this evaluation into action, they progress into the action stage.

To help an individual through the process of decisional balance, researchers and clinicians can help individuals increase their awareness of the benefits of changed behavior using motivational interviewing (MI). MI is a therapeutic technique that assesses individuals' willingness to change (i.e., how important they think change is), their ability to change (i.e., their confidence in being able to change), and their readiness for change (i.e., if they feel change is a priority). Through the MI approach, clinicians are encouraged to engage in collaborative discussions about change with their clients by evoking their reasons for change and honoring their autonomy (Butterworth, 2008; Hall et al., 2012). MI has been shown to be an effective way to promote healthy behaviors and treatment adherence for a variety of health conditions (Butterworth, 2008; Gance-Cleveland, 2005; Gearing et al., 2014; Hall et al., 2012). MI could be introduced into the *BNBD-NDD*^*TM*^ program to potentially increase participants' motivation, readiness for change, and engagement by incorporating a pre- and/or mid-intervention phone call to assess motivation and engage with participants in decisional balance.

Other potential strategies to help increase motivation and engagement that have been supported through previous research studies include pre-intervention orientation meetings (Gearing et al., 2014), between-session reminders and notifications (especially personally tailored messages; Gearing et al., 2014; Oakley-Girvan et al., 2021), and providing participants with the opportunities to see and interact with their own data (Oakley-Girvan et al., 2021).

The third research objective of the current study involved exploring the use of and satisfaction with levels of support in the *BNBD-NDD*^*TM*^ program, specifically in relation to engagement level. These supports were added into the implementation study based on feedback from the RCT. Previous research shows mixed results on the benefits of coaching and other virtual supports in eHealth interventions. For example, several studies have found evidence for improved engagement as result of virtual coaching, as well as increased participant self-efficacy and enhanced health outcomes (Hurmuz et al., 2022; Kang et al., 2021; Mohr et al., 2011; Obro et al., 2021). In contrast, other researchers have noted potential challenges with virtual coaching, such as privacy concerns, quality of coaching (i.e., coaches' competency and training to provide correct, helpful suggestions; Lentferink et al., 2017), misalignment with participant needs, and difficulty with building and maintaining appropriate relationships (Brandt et al., 2018). Further, there is a lack of guidance in the research literature around how to effectively implement virtual coaching into eHealth interventions (Mohr et al., 2011).

Similarly, in the current study, there were mixed perspectives about the levels of support available to participants. Participants in the self-guided program group (Level 1), regardless of engagement level, perceived that these supports would be helpful; however, most participants in the coach and virtual hub groups (Levels 2 and 3) did not take advantage of the supports when they were available. It appears that participants perceived that these supports would be helpful to them, but when it came to using them, they were not needed, especially by those in the Clinical Engagement group. When the parent was engaged, the program itself seemed to be enough and the addition of a coach and/or virtual hub did not seem to be necessary. In contrast, participants in the Non-clinical and No Engagement groups tended to be more in favor of these extra supports. This study supports the notion that there is mixed evidence regarding the true benefit of virtual coaching and other supports in eHealth interventions, and more research specifically in this area is needed to ensure that participants in any eHealth intervention can receive high quality, useful supports.

Other recommendations for the use of supports in the program have also been identified. One common response by participants was that they were unaware of their access to the online coach and/or virtual hub. The *BNBD-NDD*^*TM*^ research team tried to make this access well-known and obvious; however, some participants seemed to have missed this information. Future research directions should consider how to increase the awareness and knowledge of these supports, both pre-intervention as well as throughout the use of the intervention.

The fourth and final research objective of the current study was to understand the possible impacts of the COVID-19 pandemic on engagement in the program. While the interviews for this study took place after the height of the pandemic, during a time in which Canada experienced a relaxation in COVID-19-related restrictions (e.g., masking, social distancing), it is possible that the lingering effects of the pandemic played a role in impacting participants' engagement in the program over the previous months (which would have had higher levels of restriction). Due to the rise of the COVID-19 pandemic in 2019 and 2020, use of platforms such as Zoom and Microsoft Teams have increased upwards of 350% (Williams, 2021). While there are certainly positive outcomes of these platforms, including the convenience of meeting with others virtually, the excessive usage of these platforms have also led to the new phenomenon called “Zoom fatigue” (Nesher Shoshan and Wehrt, 2022). “Zoom fatigue” refers to the personal, professional, and psychological demands of using virtual technology in place of face-to-face meetings. These demands include a reduced ability to interpret body language or cues, inability to relax into natural conversation, long (and often back-to-back) meetings, unintentional encouragement of distraction and multitasking, and difficulty with blending work and home (Nesher Shoshan and Wehrt, 2022; Williams, 2021). As result of “Zoom fatigue,” individuals may be weary of using virtual technology and would rather attend meetings and appointments in person. “Zoom fatigue” may also have played a role in the engagement levels of the participants in the current study. Participants reported a variety of perspectives related to the impact of the pandemic. Some participants did not identify any impacts, while others expressed positive impacts, including increased time and flexibility to complete the program. In contrast, other participants identified negative impacts of the pandemic, including long-term illness and challenges related to routines and scheduling. At this time, it does not appear that the COVID-19 pandemic negatively impacted engagement in the *BNBD-NDD*^*TM*^ program for most participants; however, the prolonged effects of the pandemic, such as “Zoom fatigue,” should still be considered in the design of eHealth interventions.

There are several strengths and limitations of the current study that are important to consider. One significant strength of this study is the use of in-depth, qualitative data to gain a rich understanding of participants' experiences in the *BNBD-NDD*^*TM*^ program. Many of these perspectives likely would not have been accurately described using only quantitative measures. Further, this study included the perspectives of participants in the No Engagement group. Often, those who are not engaged in programs are not included in evaluation of said programs; however, in the current study, these participants were able to provide important information regarding their lack of engagement. Additionally, the current study aimed to explore various mediating factors related to engagement in the *BNBD-NDD*^*TM*^ program, such as the COVID-19 pandemic and use of the virtual supports, which helped provide a richer understanding of participants' engagement.

In terms of limitations, one possible limitation of this study is the lack of quantitative data. A mixed methods approach that carefully synthesizes and integrates qualitative and quantitative data would be helpful to better understand participants' experiences in the *BNBD-NDD*^*TM*^ program, and future research should consider this design approach. Another limitation in the current study is that all data were retrospective. While this is necessary to understand participants' experiences in the program as a whole, it would be helpful to gather data from parents before and/or during their engagement in the program to assess their readiness for change and motivation level at that time. Lastly, one of the biggest limitations to this study was the lack of examination of several possible social determinants of health and their impact on engagement. Several social determinants of health may have impeded engagement in the program, such as cultural and religious factors, parenting values, socioeconomic status and work schedules, and parental childhood experiences (Latulippe et al., 2017; Moghaddasi et al., 2017; Weisenmuller and Hilton, 2021). One important factor to consider is the heritability of NDD (e.g., ADHD is highly heritable, meaning that there is a high chance parents participating in the program may have similar challenges as their children; Faraone and Mick, 2010; Larsson et al., 2014). As such, future research on engagement levels of participants in eHealth interventions should take care to consider a wide range of social determinants of health and their possible impact on engagement, and include strategies to engage a diverse range of participants (e.g., for parents who may also have ADHD, incorporating more repetition into the intervention and providing more organizational/planning support may be helpful; Chronis-Tuscano et al., 2017).

In summary, the results from this study provide a rich, comprehensive understanding of participants' engagement in the *BNBD-NDD*^*TM*^ program related to their motivation and readiness for change. This study also provides important considerations for future research to optimize uptake and adherence to the program and improve the program's implementation and sustainability. This will ultimately lead to a more effective parent-based intervention that can help children with NDD sleep better.

## Data Availability

The datasets presented in this article are not readily available because it is qualitative data collected through interviews. Requests to access the datasets should be directed to Penny Corkum, penny.corkum@dal.ca.
